# Stem cell therapy targeting the right ventricle in pulmonary arterial hypertension: is it a potential avenue of therapy?

**DOI:** 10.1177/2045893218755979

**Published:** 2018-02-26

**Authors:** Fanny Loisel, Bastien Provost, François Haddad, Julien Guihaire, Myriam Amsallem, Bojan Vrtovec, Elie Fadel, Georges Uzan, Olaf Mercier

**Affiliations:** 1 36705 Research and Innovation Unit, Inserm UMR-S 999, Marie Lannelongue Hospital, Universite Paris Sud, Paris-Saclay University, Le Plessis Robinson, France; 2 Inserm 1197 Research Unit, Universite Paris Sud, Paris-Saclay University, Villejuif, France; 3 Cardiovascular Medicine, Stanford Hospital, Stanford University, CA, USA; 4 Department of Cardiology, Advanced Heart Failure and Transplantation Center, University Medical Center Ljubljana, Ljubljana, Slovenia; 5 Department of Thoracic and Vascular Surgery and Heart-Lung Transplantation, Marie Lannelongue Hospital, Universite Paris Sud, Paris-Saclay University, Le Plessis Robinson, France

**Keywords:** progenitor cells, stem cells, congenital heart defect, right ventricle failure, pulmonary arterial hypertension

## Abstract

Pulmonary arterial hypertension (PAH) is an incurable disease characterized by an increase in pulmonary arterial pressure due to pathological changes to the pulmonary vascular bed. As a result, the right ventricle (RV) is subject to an increased afterload and undergoes multiple changes, including a decrease in capillary density. All of these dysfunctions lead to RV failure. A number of studies have shown that RV function is one of the main prognostic factors for PAH patients. Many stem cell therapies targeting the left ventricle are currently undergoing development. The promising results observed in animal models have led to clinical trials that have shown an improvement of cardiac function. In contrast to left heart disease, stem cell therapy applied to the RV has remained poorly studied, even though it too may provide a therapeutic benefit. In this review, we discuss stem cell therapy as a treatment for RV failure in PAH. We provide an overview of the results of preclinical and clinical studies for RV cell therapies. Although a large number of studies have targeted the pulmonary circulation rather than the RV directly, there are nonetheless encouraging results in the literature that indicate that cell therapies may have a direct beneficial effect on RV function. This cell therapy strategy may therefore hold great promise and warrants further studies in PAH patients.

## Introduction

Despite significant advances in terms of its medical management, pulmonary arterial hypertension (PAH) remains an incurable condition that requires a lung or a heart and lung transplantation when refractory right heart failure occurs. It is well-known that right ventricular function is the main prognostic risk factor in PAH.^1^ Although stem cell therapies targeting the left ventricle (LV) are proving to be promising therapies in animal models, the results of recent multicenter trials with humans have been mixed.^2^ The promising results observed in animal models led to clinical trials in adults that have shown improvements in regard to LV function, infarct size, and cardiac remodeling.^3,4^ Hence, in light of the increased relevance of the pathophysiology of right ventricle (RV) failure, one can hope that the next decade will see new therapeutics that specifically target the RV.

In contrast to left heart disease, stem cell therapy applied to the RV has not been studied much, despite indications that it may be a viable therapeutic option. This review focuses on stem cell therapy as a treatment for RV failure in PAH.

## Pathophysiology of right ventricle failure

**Fig. 1.**
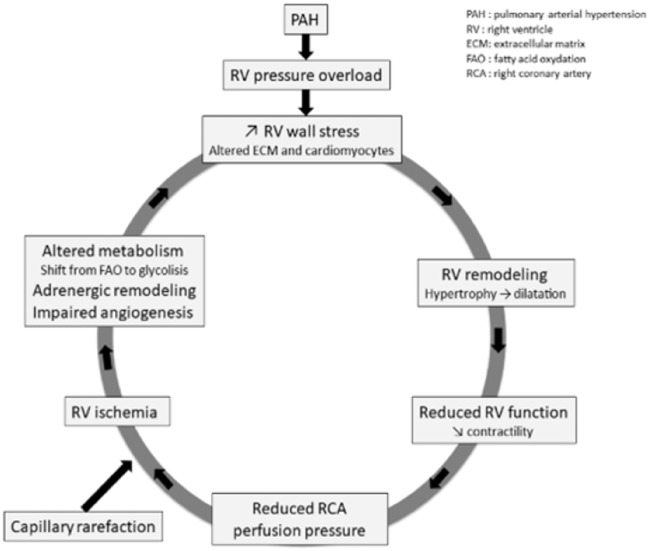
Cycle of right ventricle failure in pulmonary hypertension.

PAH is defined by the World Health Organization as pre-capillary pulmonary hypertension with a mean pulmonary arterial pressure (mPAP) ≥ 25 mmHg.^5^ PAH affects an estimated 15–60 individuals out of a million.^5,6^ It is characterized by a progressive increase in pulmonary vascular resistance (PVR) and vascular remodeling, with ensuing right heart failure and early death when it goes untreated.^7^ RV function is the main prognostic factor in PAH.^8^ Pathobiological changes in the pulmonary vascular bed are characterized by intimal hypertrophy, adventitial remodeling, smooth muscle hypertrophy, in situ vasoconstriction, and thrombosis. All of these changes increase PAP, which in turn induces a rise in pulmonary arterial resistance. As a result, the RV is subject to an increased afterload and it has to pump blood more intensely to meet the oxygen demands of the body.^9^ RV remodeling changes from an adaptive to a maladaptive phenotype as the disease progresses. The RV first undergoes an increase in wall thickness and contractility so as to maintain a normal cardiac output and ventriculo-arterial (VA) coupling. This compensatory state is called adaptive RV remodeling. As the PAH deteriorates, the RV remodeling shifts from an adaptive to a maladaptive state that includes RV dilatation, decreased contractility, and VA uncoupling (Fig. 1).^10,11^ Ultimately, patients present with end-stage RV failure that leads to death. Numerous studies have shown that survival correlates with RV function.^12^

**Fig. 2.**
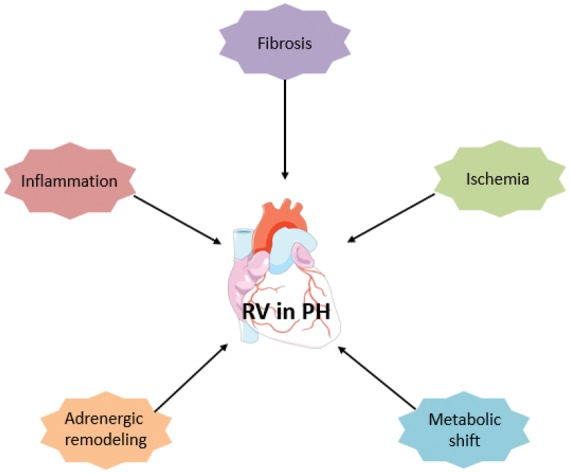
Right ventricle remodeling in pulmonary hypertension.

The shift from an adaptive to a maladaptive state is not well understood at present. RV remodeling depends on many factors including neurohormonal activation, myocardial metabolism, myocardial perfusion, genetic factors, inflammation, and alteration of the extra-cellular matrix (ECM) (Fig. 2).^11^ Unlike adaptive remodeling, maladaptive RV remodeling is characterized by dilatation, decreased cardiac output, VA uncoupling, and a high mortality rate. At the cellular level, maladaptive RV remodeling is associated with decreased angiogenesis, increased fibrosis, metabolic changes, and dysregulation of the autonomic nervous system.^13^ Although it is a poorly understood process, it appears that the transition from adaptive to maladaptive RV remodeling is a continuum that ultimately leads to end-stage heart failure. A decrease in ventricular capillary density has been found in various PAH animal models and in humans at the stage of maladaptive remodeling or heart failure.^14,15^ A degree of RV ischemia in patients suffering from PAH was first shown by Gomez et al. in 2001 using myocardial scintigraphy, and it correlated with the hemodynamic severity and the RV end-diastolic pressure.^16^ Using the monocrotaline (MCT) rat model of PAH, Sutendra et al. also found that the transition from adaptive to maladaptive RV was associated with a decrease in VEGF, myocardial SDF1 levels, and RV capillary density.^17^ Pathological examination of the RV of PAH patients has confirmed these findings, revealing a decrease in capillary density compared to a control group.^15^

Although RV failure is the main cause of mortality in PAH, it has been shown to have a tremendous capacity to reverse the remodeling when the afterload is removed, as seen following pulmonary transplantation.^18^ Plasticity is one of the key differences in the pathophysiology of RV and LV failure. Several studies have highlighted the role of myocardial ischemia as one of the leading causes of RV failure in PAH patients.^17^ Even though the mechanisms leading to decreased RV perfusion in PAH have not been entirely elucidated, we believe that targeting angiogenesis in the RV myocardium appears to be a promising approach to improve its function. The use of stem cells as therapeutic agents could, hence, represent a new treatment option for PAH. Thus, while there are no stem cell therapies at present that target the RV in PAH, studies in this regard that are focused on the RV in PAH are clearly warranted.

## Stem cells for right ventricle therapy

Given the nature of the pathophysiology of PAH induced by RV failure, the stem cells should ideally be readily available, immuno-compatible, proangiogenic, and anti-inflammatory. Of note, several cell types have already been evaluated for PAH treatment targeting the pulmonary circulation, although the RV has so far not been directly targeted.

### Mononuclear stem cells

**Fig. 3.**
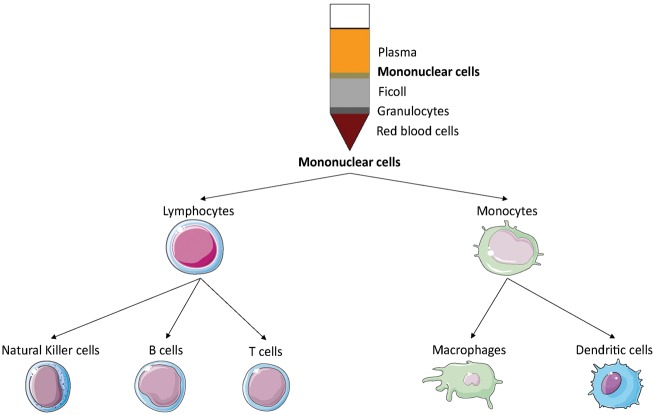
Mononuclear cell isolation allows the recovery of different cell types such as natural killers, B cells, T cells, macrophages, and dendritic cells.

Umbilical cord blood (UCB) is a readily available source of stem cells, such as mononuclear stem cells (MNC). MNC can readily be isolated from UCB by Ficoll density gradient centrifugation.^19^ UCB hence represents a promising source of cells for proangiogenic therapies. An MNC fraction isolated from UCB has been shown to exhibit pericyte-like function: they acted as proangiogenic mural cells by supporting endothelial progenitor cell (see below) network formation *in vitro*.^20^ These are the two vascular cell types required for stable microvessel formation.^21^ UCB-MNC also have antifibrotic and proangiogenic potential *in vivo*.^22^ Given that MNC comprise many different cell types (Fig. 3), determining which cell type is responsible for a potential beneficial effect is a challenge. UCB is currently the most prevalent available source for stem cells.^23^ However, it is an allogenic source (unless the cells are derived from matched samples harvested at the time that the individual was born), which is a significant limitation for its use.

Most studies of MNC to date have used UCB-derived MNC (UCB-MNC). Cantero Peral et al. have demonstrated that RV epicardial injection of autologous UCB-MNC in piglets is safe and feasible. Indeed, there were no significant cardiac injuries caused by the injection, as measured by cardiac troponin T levels and the absence of arrhythmias.^24^ In a chronic RV volume overload model, Yerebakan et al. showed that epicardial UCB-MNC injection improved RV diastolic function and increased capillary density within the area of the cell injection.^25^ Moreover, in a pulmonary artery banding murine model, UCB-MNC injections led to improvement in RV structure and function, reduction in RV fibrosis, and an increase in angiogenesis biomarkers.^22^ Lastly, in an ovine model of RV training, epicardial UCB-MNC injections improved diastolic and systolic RV functions.^26^

### Mesenchymal stromal cells

**Fig. 4.**
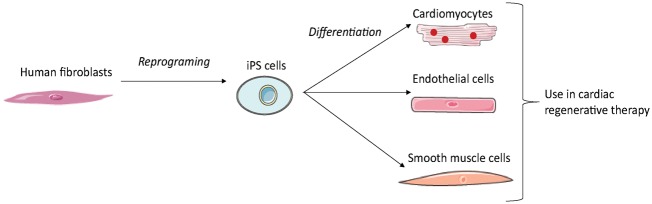
Mesenchymal stem cell isolation. Mesenchymal stem cell can be isolated from various tissues such as bone marrow, muscle, placenta, umbilical cord, or adipose tissue.

Mesenchymal stromal cells (MSC) can be isolated, for example, from bone marrow (BM), umbilical cord Wharton's jelly, placenta, adipose tissue, and muscle (Fig. 4). These multipotent stromal cells can differentiate into a variety of cell types including osteoblasts, chondrocytes, and adipocytes. They express cell surface markers such as CD73, CD90, and CD105 and they lack surface expression of CD31, CD14, CD24, and CD45.^27^ MSC have the ability to migrate to injured lung tissue where they secrete angiogenic (VEGF), anti-apoptotic (Bcl-2), and anti-inflammatory factors (IFN, IL-10, VEGF, and HGF).^28^ In normal or hypoxic conditions, they secrete factors that stimulate endothelial cell chemotaxis and adhesion.^29^ The immune tolerance of MSC is an important feature that makes them very suitable for clinical use.^30^ Given their proangiogenic and cytoprotective effects due to the release of paracrine factors, MSC are attractive candidates for RV cell therapy in PAH. They may in fact increase capillary density and protect cardiomyocytes from hypertrophy and fibrosis. Last but not least, their immunomodulatory properties make them particularly attractive for stem cell therapy.^31^ However, the properties of MSC as well as the effectiveness of their isolation can vary depending on the tissue of origin.^32^

It has been demonstrated that intravenous injection of BM and UCB-derived MSC improved right ventricular hypertrophy (RVH) and right ventricular ejection fraction (RVEF) of MCT-induced PAH rats.^33,34^ A decrease in the RV to LV + septal weight ratio after MSC injection was observed in a model of overflow-induced PAH as well.^35^ The use of human embryonic stem cell-derived (hESC) MSC yielded better results compared to adult BM-MSC in terms of the improvement of RVH and right ventricular systolic pressure (RVSP) of intravenously injected MCT-induced PAH rats.^36^ Lastly, similar results have been reported for intratracheal administration.^37^

Kanki-Horimoto et al. intravenously administered MSC overexpressing eNOS in MCT-induced PAH rats. They found that the RVSP and RV/body weight were lower in the MSC/eNOS group than in the MSC control group.^38^ Another study used MSC expressing heme oxygenase-1 (HO-1), which is known to have protective function and to restore homeostasis in many diseases. Intravenous injection of MSC expressing HO-1 in a chronic hypoxia-induced PAH model resulted in normal RVSP and a significant reduction of RVH.^39^ Kang et al. intravenously injected MSC primed with a factor that sensitized the stem cells to a chemotactic gradient in MCT-induced PAH rats. Their study revealed that primed MSC were better than human umbilical cord blood-derived non-primed MSC.^40^

It should be pointed out that we have only reported results regarding the RV. All of the studies were designed to treat pulmonary circulation, and they showed an improvement in lung injury, thus suggesting that MSC may be a promising therapeutic option for PAH. However, whether the improvement of RV function was due to a direct effect of the cell therapy or the decrease in pulmonary resistance remains an unanswered question. The RV has never been directly targeted by intravenous MSC administration, except in the setting of congenital heart defect (CHD) models. Indeed, intracoronary administration of MSC in a CHD model showed stabilization of right ventricular distolic pressure (RVDP) and RVSP after the treatment.^41^

Furthermore, as MSC have beneficial effects on endothelial progenitor cells (EPC), their co-injection allowed for the formation of a more developed vascular network than with either MSC or EPC alone.^42^ As a result, it may be interesting to administrate MSC in combination with EPC in a PAH or CHD model to see whether this results in a greater benefit.

### Endothelial progenitor cells

Endothelial progenitor cells (EPC), first described by Asahara et al. in 1997, are a circulating, BM-derived cell population. Asahara et al. found that mononuclear blood cells isolated from human peripheral blood expressing CD34 + or vascular endothelial growth factor receptor-2 (VEGFR-2) could differentiate into endothelial cells.^43^ EPC can be isolated from peripheral blood, UCB or from BM. One method for identifying EPC is based on the expression of various protein markers on the cell surface. There is no single specific marker for identifying EPC. The first markers used to identify EPC, as defined by Asahara, were CD34 and VEGFR-2. Currently, in order to be defined as EPC, the cells must express a set of distinguishing markers, while they also share common markers with hematopoietic stem cells (HSC) and circulating endothelial cells (CEC) (Table 1). This is why other characteristics are required to define cells as EPC, such as specific phenotypic and functional properties including the formation of tubular structures^44^ and their ability to migrate in response to stimuli (e.g. VEGF, SDF-1).^45^

**Table 1.** Shared markers between HSC, CEC, and EPC.

Circulating cells	Phenotype
Hematopoietic stem cells (HSC)	CD34+, CD45+, CD133+, KDR+
Circulating endothelial cells (CEC)	CD34+, CD45−, CD133−, KDR+, CD146+, CD31+, vWf+
Endothelial progenitor cells (EPC)	CD34+, CD45−, CD133+, KDR+, CD14−, CD146+

**Fig. 5.**
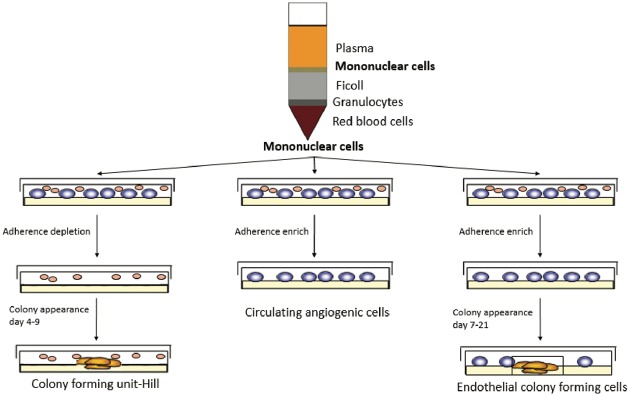
Endothelial progenitor cell isolation. A Percoll gradient allowed to isolate mononuclear cells. These cells were cultivated in an endothelial based medium. Colony forming unit-Hill appeared 7–21 days after depletion of cells in suspension.

EPC can be divided into three subpopulations: colony forming unit Hill (CFU-Hill); early outgrowth; and late outgrowth EPC (Table 2 and Fig. 5). All of these are inducers of angiogenesis, either as result of their ability to repair damaged vessels and to generate of neo-vessels or by their release of paracrine factors that stimulate vascular repair and regeneration.^46^ EPC subpopulations exhibit differences in their capacity to induce angiogenesis.^47–49^ Hence, there is a need for a clear definition of the subpopulation that is used in order to analyze and compare the results of studies of EPC therapy. Late EPC, also called endothelial colony forming cells (ECFC),^50^ can differentiate into specialized endothelial cells in the presence of appropriate stimuli.^51^ Hence, ECFC can repair damaged vessels or create new vessels.^52–54^ They can also release paracrine factors involved in angiogenesis.^55^ Consequently, EPC appear to be good candidates for potential RV-targeted cell therapy in PAH. However, it should be kept in mind that, aside from a degree of controversy regarding their definition, isolation of adult EPC from peripheral blood does not yield enough cells for stem cell therapy. They need to be expanded in culture for several weeks in order to yield enough cells to provide a potential beneficial effect in PAH patients. Isolation of EPC from UCB yields considerably more EPC than is the case for adult blood, and these cells are fully functional.^56^ However, since EPC cells are allogenic, their potential immune rejection needs to be given due consideration. At last, there is a lack of identification of the type of EPC making studies' conclusions hard to compare and translate to clinical practice.

**Table 2.** Different types of EPC (EPC can be subdivided into early and late EPC).

	Early EPC		Late EPC
Types of endothelial progenitor cells	Colony forming unit-Hill (CFU-Hill)	Circulating angiogenic cells (CAC)	Endothelial colony forming cells (ECFC)
Markers	CD34+, CD31+, CD45+, KDR+	CD34+, CD31+, CD45+, KDR+	CD34+, CD31+, CD45-, KDR+, CD144+, vWf+
Origin	Hematopoietic cells	Hematopoietic cells	Endothelial cell
Properties	Do not proliferate but express paracrine factors	Participate to angiogenesis through secretion of paracrine factors. Do not incorporate into vasculature and do not differentiate into endothelial cells	Participate to angiogenesis through differentiation into mature endothelial cells and incorporation into new vessels

Over the years, there have been many studies involving administration of autologous EPC as a treatment for PAH. However, there has been no consensual definition of EPC and the authors did not specifically target the RV in these studies.

Yip et al. observed a decrease in RVSP and RVH following intravenous tail vein injection of autologous BM-derived EPC in MCT-induced PAH rats.^57^ Xia et al. similarly obtained positive results with EPC that were isolated from blood donors and injected into MCT-induced PAH nude rats.^58^ Mirsky et al. injected CFU-Hill EPC into the tail vein, and they found no beneficial effect on MCT-induced PAH rats. They did not find cells in the lungs and the CFU-Hill EPC had low angiogenic capacities, which may explain these disappointing results.^59^ In the study by Ormiston et al., early EPC rather than late EPC were able to prevent an MCT-induced increase in RVSP and RVH. Early EPC prevented PAH through an immune-dependent mechanism due to the production of anti-inflammatory cytokines.^60^

Others have produced engineered EPC that either secrete prostacyclin^61^ or endothelial nitric oxide synthase (eNOS),^62^ which are strong pulmonary vasodilators. In both cases, engineered EPC, injected into the jugular vein of MCT-induced PAH rats, were more effective than control EPC in decreasing RVH and RVSP.

The combination of EPC with PAH drug therapies has also been studied. In MCT-induced PAH rats, EPC and sildenafil (a phosphodiesterase 5 inhibitor)^63^ or cilostazol (a phosphodiesterase 3 inhibitor)^64^ were effective in limiting the progression of PAH and they were more effective than EPC alone in attenuating RVSP and RV weight in MCT-induced PAH rats.

These results indicate that EPC may have a beneficial effect on the RV in animal models of PAH. However, the RV was not directly targeted and, as the lung circulation was the main target, the authors did not search for EPC in the heart. As a result, there is no way to know whether the beneficial effects observed on the RV were a direct effect of the injected stem cells or whether these were because the stem cells induced an improvement in the pulmonary vascular disease. However, EPC appear to have a beneficial effect on pulmonary microvascular disease as all of the studies demonstrated improvements in pulmonary hemodynamics, the number of alveolar sacs and arterioles, and pulmonary microvessel thickening.

### Cardiac progenitor cells

Cardiac progenitor cells (CPC) are a heterogenous group of cells identified in adult heart; and they are characterized by the expression of c-Kit, Sca-1, and SSEA-1. The therapeutic potential of CPC has already been documented for myocardial infarction (MI).^65^ They are thought to stimulate cardiomyocytes and vascular cell transdifferentiation, as well as secretion of paracrine factors that promote neovascularization and activation of endogenous CPC.^66^ These properties could be beneficial to the RV in PAH by causing an increase in capillary density and by activating the production of “efficient” new cardiomyocytes. However, adult CPC isolation is an invasive procedure as these cells are isolated from the patient's heart tissue. On the other hand, although the use of embryo-derived CPC raises ethical issues, these can be circumvented by the use of autologous iPS cells (see below). Lastly, CPC include a large population of different cell types,^66^ which makes it difficult to determine the one that will prove to be the most efficient. Similar to EPC, a clear identification and characterization of the cells used are crucial to discuss the results of studies.

In addition to CPC, cardiosphere derived cells (CDC) are an option, and they can be obtained from adult human biopsies. Explants are plated and the outgrowth cells derived from them self-assemble into cardiospheres that yield CDC once replated.^67,68^ Recent studies have shown that exosomes released from human CDC are cardioprotective. They have been shown to inhibit apoptosis, promote cardiomyocytes proliferation, and enhance angiogenesis by secretion of paracrine factors.^69,70^ In vivo, CDC administration in pigs with acute MI reduced the number of apoptotic cardiomyocytes in the infarct border zone and scar, decreased fibrosis, and increased vessel density. Thus, they can enhance left ventricular function by contributing to tissue repair. These results indicate that CDC can inhibit cardiomyocyte death after MI, thereby providing a degree of cardioprotection, while they can also confer long-lasting functional benefits for up to two months.^71^

CDC were tested clinically in the CADUCEUS trial (Cardiosphere-Derived Autologous Stem Cells to Reverse Ventricular Dysfunction), which examined the safety and efficacy of intracoronary autologous administration of CDC in patients with LV dysfunction after MI. The administration of CDC did not lead to significant safety issues and it resulted in decreased infarct scar size and improved LV function at one year post treatment.^67^ Moreover, the phase I of the ALLSTAR trial (ALLogeneic Heart STem Cells to Achieve Myocardial Regeneration), which included MI patients, showed that intracoronary infusion of allogeneic CDC is safe, with minimal or no discernible immune reactions. The phase II trial will compare intracoronary CDC to a placebo in 120 patients.^72^ These findings suggest that exosomes from allogeneic CDC can be safely used in clinical applications. As for CPC isolation, CDC isolation requires heart explants and it is hence an invasive procedure.

Ischemic heart disease is associated with loss of cardiomyocytes, ultimately leading to pump failure in MI patients.^73^ The use of CPC or CDC as stem cell therapies could prove to be useful to prevent cardiomyocyte apoptosis and fibrosis. To our knowledge, cardiomyocyte apoptosis has not been reported in PAH RV failure so far. Consequently, it is not clear whether using CPC or CDC in RV failure in PAH is relevant. However, this could nonetheless still be useful to reverse fibrosis and to increase angiogenesis in the dysfunctional right ventricle.

The safety and feasibility of epicardial administration of human embryonic stem cell–derived CPC were assessed in a pig model mimicking RV volume overload of tetralogy of Fallot. Animals receiving cell therapy exhibited a decrease in RV fibrosis that did not lead to an improvement of the RV dysfunction observed in this model. None of the administered cells could be found in the myocardium. Given that these were human cells, immune rejection may have occurred. It is also possible that the hypoxic environment of the grafted cells affected CPC survival.^74^

### Adipose-derived stem cells

Adipose-derived stem cells (ADSC) can readily be isolated from white adipose tissue by liposuction and they are suitable for autotransplantation.^75^ ADSC have wound healing potential as result of their ability to secrete angiogenic and anti-apoptotic growth factors such as granulocyte–macrophage colony-stimulating factor, VEGF, hepatocyte growth factor (HGF), basic fibroblast growth factor (bFGF), and TGF-a.^76^ ADSC can promote angiogenesis in models of hind limb ischemia by undergoing differentiation into endothelial cells which are incorporated into the walls of newly formed vessels and by secretion of paracrine factors.^77,78^ Nagata et al. compared ADSC isolated from subcutaneous, visceral, cardiac, and subscapular adipose tissues of mice, and they showed that the ADSC isolated from cardiac adipose tissue, when administered in a mouse MI model, proliferated better, while they also had the highest cardiac functional recovery and the highest rate of recruitment to ischemic myocardium. In fact, these cells had the potential to differentiate into cardiovascular lineage cells such as cardiomyocytes, endothelial cells, and vascular smooth muscle cells.^79^ Moreover, a phase I clinical trial of ADSCs has been conducted in patients with ischemic cardiomyopathy (PRECISE trial) to examine safety and feasibility, and it revealed significant improvements in left ventricular mass and a reduction of ischemia.^80^ Consequently, ADSC appear to be a good choice of stem cells to treat the RV in PAH as, in addition to being autologous, plentiful, and readily obtainable, they can induce angiogenesis without undergoing apoptosis.

The use of ADSC in MCT-induced PAH rats has been shown to improve pulmonary vascular remodeling and to decrease RVH.^81^ Moreover, ADSC transduced to express cyclooxygenase-1 have been shown to attenuate MCT-induced PAH and RVH.^82^

### iPS derived cells

**Fig. 6.**
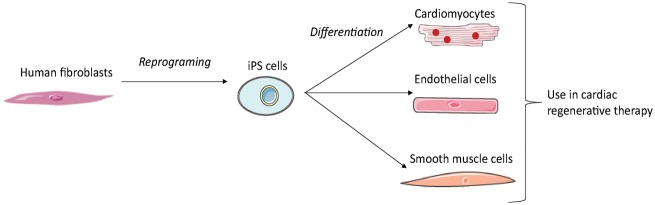
Generation of cell derived iPS for cardiac regenerative therapy. The reprogramming of adult fibroblasts thanks to a combination of specific factors leads to generation of induced pluripotent cells. These cells can be differentiated into the desired cell type for cardiac regenerative therapy.

In 2006, Takahashi and Yamanaka demonstrated that it was possible to induce pluripotent stem cells from mouse embryonic or adult fibroblasts using a combination of specific factors. These cells are known as induced pluripotent stem (iPS) cells.^83^ The generation of cardiomyocytes, endothelial cells, or smooth muscle cells from human iPS requires three steps: reprogramming of human fibroblasts into iPS; differentiation of iPS into the desired cell type; and purification and transplantation of these iPS-derived cells (Fig. 6). As several hundred million cells are required for cardiac regenerative therapy, an efficient method of production is needed. However, preparing pure cardiomyocytes without a purification step entails a risk of contamination by tumor-initiating cells.^84^ Interestingly, intramyocardial transplantation of human iPSC-derived cardiomyocytes, endothelial cells, and smooth muscle cells into a pig MI model showed that human iPSC-derived cardiomyocytes can integrate into host myocardium and generate organized sarcomeric structures, while endothelial and smooth muscle cells contribute to the host vasculature. This trilineage cell transplantation can significantly improve left ventricular function and angiogenesis, while reducing infarct size and cell apoptosis.^85^ iPS may hence provide a useful source of patient-specific cells, as they can be isolated from their own dermal fibroblasts.

The benefits of iPS cell transplantation in MCT-induced PAH rats on the hemodynamic function of the RV were assessed in the study by Huang et al. Tail vein administration of murine iPS cells was performed once per week for a period of one month. A significant decrease in RVH, RVSP, and lung tissue inflammation were observed.^86^ However, due to their embryonic stem cell-like properties, iPS cell are tumorigenic.^87^ Hence, to overcome the problem of tumorigenesis, in a lot of studies the iPS cells were made to differentiate *in vitro* into cardiomyocytes, or other cell types, before being transplanted into the animal model hosts. As there was no mention of tumor formation in study by Huang et al., the use of undifferentiated iPS cells may in fact be inconsequential in this regard. Thus, the administration of iPS derived from cardiomyocytes and endothelial cells, as used in the study by Lei Ye et al.,^85^ directly into the RV in a PAH model may be a suitable approach.

**Fig. 7.**
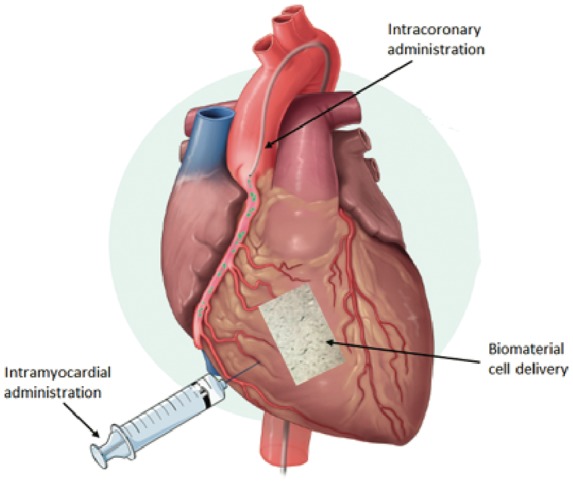
Representative markers of stem cells usable for right ventricle cell therapy.

Once again, it is important to emphasize that when stem cells therapies are not administered directly into the RV, beneficial effects on RV function may well be mediated through their action on the pulmonary unit and more precisely through the reduction of afterload. Preclinical studies of stem cell therapy for pulmonary hypertension are resumed in Table 3 and the markers of these stem cells are referenced in Figure 7.

**Table 3.** Preclinical studies of stem cell therapy for pulmonary hypertension.

Models	Cell type	Route of administration	Pulmonary effect	Direct / indirect RV effect	Found cell engraftment	Study primarily designed for RV effects	Authors
Pre clinical study							
MCT rats	EPC	Intravenous	↓connexin43, eNOS expression, ↑ alveolar sacs, ↑ small lung arterioles	Indirect. ↓RVSP, ↓ RVH, ↓ connexin43, eNOS expression	In pulmonary arterioles	No	Yip et al., 2008
			↑ n small lung arterioles, ↓ pulmonary arteriole muscularization	Indirect. ↓RVSP, ↓ RVH	In small pulmonary arterioles	No	Xia et al., 2009
			↓ pulmonary arteriole muscularization	Indirect. ↓RVSP, ↓ RVH	In lung tissue after 15 min but not 24h after injection	No	Ormiston et al., 2009
			ø	ø	ø	No	Mirsky et al., 2011
	EPC producing prostacyclin	Intravenous	↓ pulmonary vessel wall thickening, ↓ cell proliferation in pulmonary vessel wall	Indirect. ↓RVSP, ↓ RVH	In lungs up to 25 days	No	Zhou et al., 2013
	eNOS transduced EPC	Intravenous	↓ pulmonary arteriole muscularization, ↑ microvascular perfusion	Indirect. ↓RVSP, ↓ RVH	In distal arterioles	No	Zhao et al., 2005
	EPC + sildenafil	Intravenous	↑ alveolar sacs, ↑ small lung arterioles, ↓ apoptic and inflammatory biomarkers	Indirect. ↓ apoptic and inflammatory biomarkers, ↓RVSP, ↓ RV weigh	ø	No	Yen et al., 2013
	EPC + cilostazol	Intravenous	↑ alveolar sacs, ↑ small lung arterioles	Indirect. ↓ connexin43, eNOS expression, ↓RVSP, ↓ RV weigh	In pulmonary arterioles	No	Sun et al., 2009
	MSC	Intravenous	↓ thickening of arterioles and alveolar septa	Indirect. ↓RVDP, ↓ RVH, ↓ RVEF	ø	No	Umar et al., 2009
			↓ media wall thickness, ↓ intra-acinar muscular pulmonary arteries	Indirect. ↓ RVP, ↓ RVH, ↓ mRVP	Lung tissue	No	Lee et al., 2015
			↓ media wall thickness, ↑ capillary density	Indirect. ↓RVSP, ↓RVH	Pulmonary vessels	No	Yuelin Zhang et al., 2012
		Intratracheal	↓PAP, ↓PVR	Indirect. ↓ RVH	Lung	No	Baber et al., 2006
	MSC primed with S1P	Intravenous	↓ pulmonary vascular wall thickness, ↑ capillary density	Indirect. ↓RVSP, ↓ RV/(LV+S)	ø	No	Kang et al., 2015
	MSC expressing Heme Oxygenase-1	Intravenous	↓ media wall thickness, ↓ inflammation	Indirect. ↓RVSP, ↓ RVH	Lung	No	Liang et al., 2010
	MSC overexpressing eNOS	Intravenous	ø	Direct. ↓RVSP, ↓ RV weight/body weight	ø	No	Kanki Horimoto et al., 2006
	ADSC	Intravenous	↓mPAP, ↓wall/lumen thickness, ↓wall/lumen area	Indirect. ↓RVH	Lung tissue	No	Luo et al., 2014
	ADSC expressing COX-1	Intratracheal	↓mPAP, ↓ vascular remodeling	Indirect. ↓RVH, ↓RV mass	Lung parenchyma	No	Somanna et al., 2014
	iPS cells	Intravenous	↓wall/lumen thickness, ↓lung tissue inflammation (IL-1β, IL-6, IL-12, IL-23, IFNγ)	Direct. ↓RVH,↓RVSP	Lung parenchyma	No	Huang et al., 2016
Left to right shunt rat	MSC	Intravenous	↓mPAP, ↓ media thickness, ↓ intima thickness, ↓ pulmonary arteries luminal area, ↓ inflammation factors	Indirect. ↓RV/(LV+S), ↓RVSP, ↓mRVP, ↓RV/body weight	ø	No	Luan et al.
LV pressure overload with biventricular failure in rat	MSC	Intracoronary	ø	Direct. ↓RVSP, ↓RVDP, ↓ inflammation, apoptosis and ECM remodeling markers	ø	No	Molina et al., 2008
RV volume overload piglet	CPC	Epicardial	ø	Direct. ↓ in peri-myocytes fibrosis, protection against arrhythmia	No cell survival in RV at 3 months	No	Lambert et al., 2015
RV volume overload sheep	MNC	Epicardial	ø	Direct. Improved RV diastolic function under dobutamine stress, ↑ capillary density	ø	No	Yerebakan et al., 2009

### What are the best stem cells to target the right ventricle in PAH? (Table 4)

The use of cells isolated from autologous umbilical cord blood and embryo-derived cells, such as CPC, can be subject to ethical concerns and legal restrictions, as well as immunological issues. The use of iPS may be a suitable option to overcome these issues as they are derived from somatic cells and they are genetically identical to the patient. However, their reprogramming may induce genetic changes that could lead to tumor initiation.^88,89^ For practical reasons, the cells should be readily available at the right clinical dose at the time of injection. This means that the isolated cells need to have previously undergone cryopreservation, even though this is not optimal for maintaining cell properties. Indeed, there is still a considerable need for new approaches to optimize cryopreservation.^90^ As RV failure in the context of PAH is a chronic condition, patient-specific manufacture of autologous cell products could be viewed to be more suitable approach than isolating cells from the patient in advance and freezing them. However, as with other cardiovascular pathologies, it has been shown in idiopathic PAH (iPAH) patients that the number of EPC are lower compared to healthy patients.^91^ There are three options to address this: isolation of cells from the patient before the pathology is established (from UCB or from a peripheral blood sample taken during childhood); use of an allogenic stem cells source (from a stem cell-derived UCB bank); or use of iPS cells derived from the patient's own fibroblasts.

The outcomes of preclinical and clinical studies regarding stem cell therapy for heart diseases depend on the type of cells as well as the route of administration. Finally, while promoting angiogenesis may be beneficial for improving RV function, it may be detrimental for pulmonary vascular remodeling in PAH patients. An adequate degree of care should hence be taken to choose the best cells and the best way to administer these cells to the heart so as to avoid detrimental side effects on the pulmonary circulation.^92^

**Table 4.** Comparing cell types suitable for stem cell therapy targeting the right ventricle in pulmonary hypertension patients.

Cell type	Advantages	Drawbacks	Targets
Mononuclear stem cells	- Easy to isolate - The isolation gives a lot of cells	- Include a lot of cell type	- Pro-angiogenic - Anti-fibrotic
Mesenchymal stromal cells	- Can be isolated from different tissues - Immune compatible	- Their functions can be different depending on their origin	- Pro-angiogenic - Anti-apoptotic - Anti-inflammatory
Endothelial progenitor cells	- Can differentiate into specialized endothelial cells - Their isolation is minimally invasive	- Difficult to identify - Have to be expanded in culture for a few weeks to have enough cells to administrate	- Pro-angiogenic
Cardiac progenitor cells	- Can activate endogenous CPC to promote the production of new “efficient” cardiomyocytes	- Very invasive procedure to isolate them: heart biopsies	- Pro-angiogenic - Promotion of cardiomyocyte proliferation
Cardiosphere derived cells	- Cardioprotective	- Very invasive procedure to isolate them: heart biopsies	- Pro-angiogenic - Anti-apoptotic - Promotion of cardiomyocyte proliferation
Adipose-derived stem cells	- Easy to isolate - Suitable for autotransplantation	- Invasive procedure to isolate them: liposuccion	- Pro-angiogenic - Anti-apoptotic
iPS derived cells	- Can provide a source of patient-specific cells	- Difficult to purify cells - Risk of tumor initiating cells	The effect will depend on the cell type derived from iPS cells

### Potential administration routes (Fig.8)

Intravenous delivery of stem cells is the easiest and least invasive option for administration. It is a feasible and low-risk route. However, it has been shown that the retention rate in the heart is poor with this method of administration.^93^

**Fig. 8.**
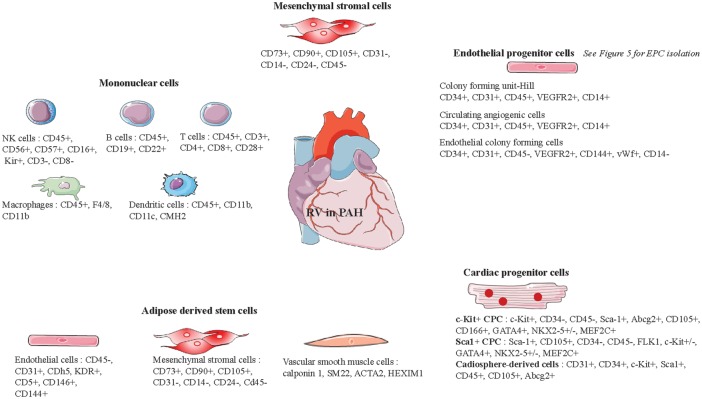
Routes of administration for stem cell therapy targeting the right ventricle in PAH.

Intracoronary delivery is one of the most commonly used methods for administration. Intracoronary injection using various cell types has already been tried and tested in several clinical trials, and have proven its safety and effectiveness. However, this technique involves a risk of coronary occlusion or microvascular obstruction by the infused cells, which can lead to myocardial infarct. Intracoronary administration appears to have a lower efficiency in terms of myocardial cell retention compared to intracardiac administration.^94^

Intracardiac delivery is the most effective and invasive route for administration.^93^ It can be divided into two subtypes: epicardial and transendocardial injection. Vrtovec et al. compared intracoronary versus transendocardial CD34 + cell delivery in patients with non-ischemic dilated cardiomyopathy, and they found that transendocardial delivery was associated with a better clinical response due to a higher level of myocardial cell retention.^95^ Although the intracardiac administration route appears to be optimal in terms of cell retention, this method is invasive and it entails a risk of ventricle wall perforation and cardiac arrhythmias.^96^

Cells are released in a new and damaged environment, as opposed to the ideal environment in which they were cultured before the injection. As a result, cell death and cell retention are two major problems that still need to be solved. To enhance cell survival and delivery, the addition of biomaterials that mimic or that are derived from ECM have undergone assessment in recent years (e.g. Matrigel®, decellularized ECM, hyaluronic acid, and collagen).^97^ The goal of this approach is to use biocompatible biomaterials as an ideal environment for cell survival that could permit continuous cell delivery to organs. The currently available biomaterial delivery systems are patches that can be applied on the surface of the organ and injectable hydrogel that can be incorporated in the organ and 3D scaffold.^98,99^

## Clinical studies targeting the right ventricle (Table 5)

Rupp et al. reported the first case of intracoronary infusion of autologous BM-derived progenitor cells in an infant of 11 months who was afflicted with hypoplastic left heart syndrome (HLHS). HLHS is a very serious type of CHD, also known as “univentricular heart syndrome,” which is characterized by hypoplasia of the LV and that ultimately leads to RV failure. Three months after administration, it was found that the RVEF had increased and that the RV function had dramatically improved.^100^ A second study was performed in nine children with severe terminal heart failure for whom a heart transplant remained the last option. These children were treated by intracoronary infusion of autologous BM-derived MNC. This study showed improvement in terms of the NYHA classification and an increase in the LVEF.^101^

**Table 5.** Clinical studies of stem cell therapy for pulmonary hypertension.

	Pathology	Patients (n)	Type of cells	Method of cell transplantation	State	Results	Authors
Clinical study							
PHACeT (phase 1) 2010	iPAH	7	eNOS-enhanced endothelial progenitor cells	PA injection	Completed	-Improvement in exercise tolerance - No hemodynamic improvement	Granton et al., 2015
TICAP (phase 1) 2012	Hypoplastic left heart syndrome	14 (7 treated and 7 control)	Cardiosphere derived cells	Direct injection in the right and the left coronary arteries	Completed	- Improved RV function at 36 months after staged cardiac reconstruction	Ishigami et al., 2015
PERSEUS (phase 2) 2018	Hypoplastic left heart syndrome	X	Cardiosphere derived cells	Direct injection in the right and the left coronary arteries	Recruiting	X	Mayo Clinic

The Mayo Clinic carried out a stem cell trial in 2014 to treat children with HLHS. This clinical trial aimed to determine the safety and feasibility of injections of autologous UCB-MNC cells into the RV of HLHS children undergoing a particular surgical procedure. The investigators sought to investigate whether autologous UCB could strengthen the RV muscle.^102^

The first case report of intraoperative intramyocardial injection of autologous UCB yielded encouraging findings, such as a progressive improvement in the RVEF over the course of the subsequent three months. However, it is not clear whether the improvement resulted from the regenerative therapy or from the surgical procedure that was performed.^103^

Wang et al. investigated the feasibility, safety, and clinical outcomes of intravenous infusion of autologous EPC in patients with iPAH. They did not specifically study RV function in these patients, although they did find a significant improvement in mPAP, PVR, and cardiac output. This preliminary study showed that transplantation of autologous EPC may be beneficial in patients with iPAH.^104^ The same type of study has been carried out in children with iPAH, and the outcome was similar: intravenous infusion of autologous EPC was feasible and associated with improvements in exercise capacity and pulmonary hemodynamics.^105^ The PHACeT trial aimed to evaluate the safety of injecting EPC transfected with eNOS in PAH patients who were refractory to PAH-specific therapies. Three deliveries of transfected EPC were performed in the right atrium in seven patients. Although once again the RV function was not studied, this trial nonetheless showed that the cell infusion was well tolerated and that the 6-min walk distance (6MWD) was significantly increased at one, three, and six months.^106^

The TICAP (Transcoronary Infusion of Cardiac Progenitor cells) Phase I clinical trial for pediatric patients with CHD, using autologous CPC, was recently concluded. Autologous CPC were isolated from seven children with HLHS and delivered by intracoronary administration. Eighteen months after the cell administration, the CPC-treated patients exhibited an improvement in RVEF and clinical status compared to the control patients who received palliation surgery without cell therapy.^107,108^

## Perspectives

To date, all of the stem cells clinical trials targeting PAH have focused on pulmonary vascular disease. Whether the observed concomitant improvement of RV function resulted from a stem cell effect rather than an improvement in pulmonary hemodynamics remains unknown. These trials have nonetheless demonstrated that transcoronary, transatrial, and intravenous stem cell administrations are feasible, safe, and reliable. To our knowledge, the only stem cell trial in regard to RV in pediatric patients with CHD showed RV function and clinical improvement. Indeed, clinical studies involving the administration of stem cells are more developed in regard to heart defects where the LV is affected, such as the SCIPIO clinical trial.^109^ This trial revealed that autologous intracoronary administration of CSC in patients with ischemic cardiomyopathy led to improvement in the overall and regional LV function. A phase II clinical trial (MSC-HF),^110^ where endocardial administration of autologous BM-MSC was performed in patients with chronic ischemic heart disease, also showed improvement in LV function. A phase I trial has assessed the safety and feasibility of the epicardial transplantation of skeletal myoblast in patients with ischemic cardiomyopathy (CAuSMIC).^111^ Vrtovec and his team employed CD34 + cells, and they studied intracoronary administration in patients who either had dilated cardiomyopathy^112^ or non-ischemic dilated cardiomyopathy.^113^ They also studied transendocardial administration in patients with ischemic cardiomyopathy.^114^ All of their studies revealed an increase in LVEF and the 6MWD, a decrease in N-terminal pro-BNP, and improved long-term survival.

Given that administration of stem cells targeting the LV has already been shown to yield clinical benefits, there is ample reason to similarly use stem cells to treat the RV. RV function is the main determinant of survival in patients with PAH.^1^ We hypothesize that reinforcing the RV rather than trying to treat the lungs in PAH could be an alternative approach to increasing patient survival.

The vast majority of LV cell therapy studies examined cells delivery after MI. Here we proposed a scenario where cells would be delivered into a maladaptive RV that is continuously exposed to a pathologically elevated PAP which is different from what is already done in the LV where cells are delivered following an acute injury. Even if the injected cells are able to promote cardiomyocyte regeneration or enhance capillary density, one may wonder if these effects will persist in the face of the persistent increased PAP. For this reason, we believe that further studies are needed to address the sustained effect of RV stem cell therapy as well as the role of repeated injections, keeping in mind that RV cell therapy should be performed as part of a multimodality treatment targeting the RV and the pulmonary circulation.

Lastly, the main mode of action of stem cells may in fact involve a paracrine effect, as it is difficult to determine whether the cells are actually incorporated into the organ. Using exosomes or cell-derived products may be a way to avoid immune reaction while still yielding similar outcomes. Promising results in this regard have been published from a study involving MCT rats.^115^

As previously mentioned, it is difficult to compare the results of preclinical and clinical trials of RV stem cell therapy as the exact nature of delivered cells remains undetermined. Hence, there is an urgent necessity to move to standardized stem cell identification before use. For instance, there is large evidence of the heterogeneity of CPC population^66^ which may have different role in cardiac regeneration. Another important point is the evaluation of the mechanism of action of cardiac cell therapy which remains understudied. Few studies have designed *in vitro* experiments to elucidate potential mechanism of action showing that the effect of EPC therapy after MI involved IGF and TGFß pathways.^116,117^ Further studies are needed to better understand how cell therapy could help the right ventricle of PAH patients.

## Conclusions

Cell therapy holds promise in terms of providing support for RV function in PAH patients. Encouraging results have been published that highlight the feasibility, safety, and efficiency of such therapeutic approaches. Further studies are needed, however, to evaluate RV stem cell therapy in addition to pulmonary vessel therapy as an established therapy before transplantation can be used to treat PAH patients.
